# Deregulation and Targeting of TP53 Pathway in Multiple Myeloma

**DOI:** 10.3389/fonc.2018.00665

**Published:** 2019-01-09

**Authors:** Katarina K. Jovanović, Guillaume Escure, Jordane Demonchy, Alexandre Willaume, Zoe Van de Wyngaert, Meryem Farhat, Paul Chauvet, Thierry Facon, Bruno Quesnel, Salomon Manier

**Affiliations:** ^1^IRCL, INSERM UMR-S1172, University of Lille, Lille, France; ^2^Department of Hematology, CHU Lille, University of Lille, Lille, France

**Keywords:** *TP53*, multiple myeloma, targeted therapy, clonal evolution, precision medicine

## Abstract

Multiple Myeloma (MM) is an incurable disease characterized by a clonal evolution across the course of the diseases and multiple lines of treatment. Among genomic drivers of the disease, alterations of the tumor suppressor *TP53* are associated with poor outcomes. In physiological situation, once activated by oncogenic stress or DNA damage, p53 induces either cell-cycle arrest or apoptosis depending on the cellular context. Its inactivation participates to drug resistance in MM. The frequency of *TP53* alterations increases along with the progression of the disease, from 5 at diagnosis to 75% at late relapses. Multiple mechanisms of regulation lead to decreased expression of p53, such as deletion 17p, *TP53* mutations, specific microRNAs overexpression, *TP53* promoter methylations, and *MDM2* overexpression. Several therapeutic approaches aim to target the p53 pathway, either by blocking its interaction with MDM2 or by restoring the function of the altered protein. In this review, we describe the mechanism of deregulation of *TP53* in MM, its role in MM progression, and the therapeutic options to interact with the *TP53* pathway.

## Introduction

Multiple Myeloma (MM) is an incurable hematological malignancy developing as a result of clonal proliferation of plasma cells originating from post–germinal-center B cells (1). Age-adjusted rates of new cases and deaths, covering the period from 2011–2015, show that new myeloma cases incidence was 6.6 per 100,000 people per year, while the number of deaths was 3.3 per 100,000 people per year (2). MM accounts for ~10% of all hematologic malignancies (3), and mostly affects elderly people, with 69 years being the median age at diagnosis (2).

One of the distinguished characteristics of MM is the clonal progression of the disease, which is reflected through precursor stages, named monoclonal gammopathy of undetermined significance (MGUS), and smoldering multiple myeloma (SMM) (4–6). Genomic events occurring across the course of the disease can be divided into primary and secondary genomic events, which altogether form a unique genomic landscape of the disease. Primary events are further divided into hyperdiploid (HRD) and non-HRD subtypes, which are mutually exclusive. Primary HRD events are usually trisomies of odd-numbered chromosomes 3, 5, 7, 9, 11, 15, 19, and/or 21 (7). Primary non-HRD events include translocations of the immunoglobulin (Ig) heavy chains (IGH), with five most frequently occurring translocations being t(11;14) (15%), t(4;14) (12%), t(14;16) (3%), t(14;20) (2%), and t(6;14) (1%), as well as del13q, which is the most frequent deletion in MM (59%) (8). All these primary events are usually detected at the MGUS stage of the disease development. Most frequent copy number gains and losses found in MM are del13q (45%), 1q+ (40%), del14q (39%), del16q (35%), del6q (33%), del1p (30%), and del8p (25%), all representing secondary mutation events, that can be also seen as driver events (9, 10).

*TP53* gene is located at the chromosome 17p13.1, coding for p53 tumor suppressor protein, which is also known as guardian of the genome. The whole p53 signaling network is turned off under the normal physiological conditions, when p53 itself is expressed at low levels. In response to cellular stress, such as hypoxia, DNA damage, heat shock, p53 pathway becomes activated by means of protein stabilization through posttranslational mechanisms, which include phosphorylation and acetylation. The resulting accumulation of p53 protein in the nucleus triggers activation of various downstream pathways, which work in cooperation to keep the genomic integrity and homeostasis of the cell. This is achieved through several tumor suppressive mechanisms, including cell cycle arrest, apoptosis, and angiogenesis inhibition (11, 12).

In general, mutations in *TP53* have been found in 50% of all human cancers (13). In MM, the frequency of *TP53* alterations—by means of mutations and deletions—are more frequent in late stages of the disease and are associated with treatment resistance (7, 14). In this review we focus on the role of deregulated p53 in the progression of MM and the latest the therapeutic approaches designed to target specifically this tumor suppressor.

## The *TP53* Tumor Suppressor Gene

Ten years after the initial discovery of p53 in 1979 as a host cell protein bound to T antigen in SV40-transformed mouse cells (15), it was finally established that this 53 kDa protein performs its role as a tumor suppressor in cell culture, contrary to the popular belief that it functions as an oncogene (16). Immediately after this discovery inactivating *TP53* mutations were confirmed to be a common event in the colorectal cancer tumorigenesis (17), and mutations occurring within this tumor suppressor were detected as a distinctive feature of Li-Fraumeni syndrome (18).

**Figure 1:**
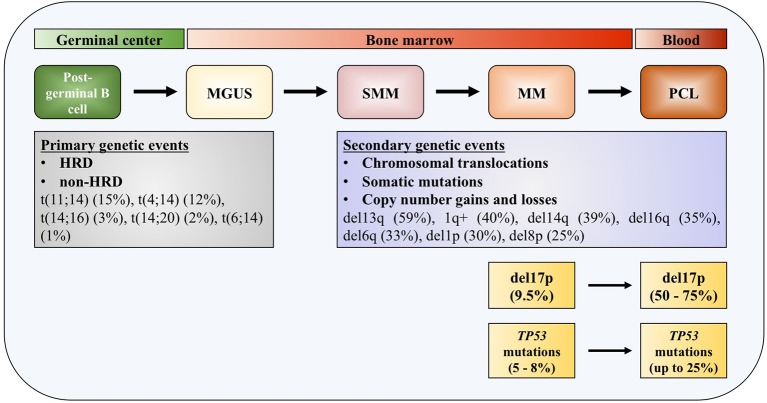
*TP53* deregulation in multiple myeloma. Increased incidence of deletion 17p and *TP53* mutations across the course of the disease. MGUS, monoclonal gamopathy of undetermined significance; SMM, smoldering multiple myeloma; MM, multiple myeloma; PCL, plasma cell leukemia; HDR, hyperdiploidy.

The p53 protein is structurally organized into several domains that are crucial for maintaining several of its functions. Two N-terminal transactivation domains, TAD1, and TAD2 respectively, are followed by a conserved proline-rich domain, which plays a role in DNA repair in response to γ-radiation (19). Positioned centrally, DNA binding domain is responsible for the site-specific DNA-binding function of p53 and represents a hot spot for the most of tumor-derived missense mutations (20). Within the C-terminal domain are located sequences necessary for nuclear localization and non-specific DNA binding, as well as an oligomerization domain mediating the formation of homo- and hetero-tetramers (21) (Figure 1).

Since p53 is a transcription factor that can sense cellular stress and gets activated in response to the DNA damage and oncogene activation, during homeostasis its levels are kept low through interaction with E3 ubiquitin-protein ligase MDM2. MDM2 works on p53 inhibition in two ways, by interacting with its transactivation domain and by targeting p53 for proteasomal degradation, conferring a very short half-life of p53 in the range from 5 to 30 min (22, 23). Moreover, MDM2 expression is regulated by p53, meaning that low levels of tumor suppressor are maintained via negative feedback loop in normal physiological conditions. Depending on the nature of the cellular stress, mechanisms of activation of p53 can be different—DNA damage results in inhibition of MDM2-mediated degradation of p53 (24), whereas oncogenic signaling activates the ARF tumor suppressor, which also prevents MDM2 from degrading p53 (25).

During the response to oncogenic stress, ATM/ATR, CHK1, and CHK2 kinases phosphorylate p53, disrupting its binding to MDM2. At the same time, CBP and KAT5 acetyl-transferases induces acetylation of the tumor suppressor, specifically at lysine 120 (K120) within the DNA-binding domain (24, 26, 27).

Once activated, p53 induces either cell-cycle arrest or apoptosis depending on the cellular context, by transactivation of its downstream target genes, such as *CDKN1A* (coding for p21 protein), resulting in cell cycle arrest and senescence, and *BAX, PUMA*, and *NOXA*, triggering apoptosis (23). Recently it was reported that p53 is also involved in metabolism regulation by decreasing expression of the cystine/glutamate antiporter XCT2 (transcribed from *SLC7A11* gene) and upregulating expression of glutaminase 2 (*GLS2* gene) (28, 29).

## Deregulation of *TP53* in Multiple Myeloma

### Deletion of 17p

Deletions of chromosome 17p13 region containing *TP53* gene are usually monoallelic and associated with less favorable outcome in patients with MM (30–32). The adverse outcome is observed in patients at diagnosis with 17p deletion affecting more than 60% of their plasma cells, which translate in shorter median event-free survival (EFS) (14.6 months) and median overall survival (OS) (22.4 months), compared to patients without this genomic aberration or in <60% of plasma cells (31). In another study, 17p deletion was also associated with worse progression-free survival (PFS) and OS irrespective of the percentage of the tumor fraction of the alteration (33). In patients newly diagnosed with symptomatic myeloma (NDMM), loss of 17p was detected with a frequency of 9.5% (34). This number increases in the most aggressive forms of the disease, up to 50% in primary plasma cell leukemia (pPCL), and even up to 75% in secondary plasma cell leukemia (sPCL) (35). Together with serum β_2_-microglobulin and serum albumin levels (the International Staging System, ISS) and serum lactate dehydrogenase (LDH) level, 17p deletion—together with t(4;14) and t(14;16)—is included in the revised International Staging System (R-ISS) (32).

### *TP53* Mutations

Most of the *TP53* mutations occurring in MM—as in human cancers—are missense mutations (Figure 1). Interestingly, in some cases it was found that protein products of missense mutant *TP53* can obtain gain of function (GOF) activities, making such gene an oncogene which is promoting tumor progression, rather than being a tumor suppressor (36–38). Around 10% of *TP53* mutations are found to be nonsense mutations resulting in truncated p53 proteins (39). Mutations in *TP53* gene are reported in generally low frequencies in NDMM by different research groups, usually around 8% (40–42). In a recent report on 1,273 patients with NDMM, it was slightly lower, around 5% (8). On the contrary, in the later stages of the disease percentage increases significantly, to 25% in PCL, indicating role of these mutations in the disease progression and drug resistance (35, 43). The presence of mutations in *TP53* gene is connected to adverse outcome in terms of PFS and OS, same as for 17p deletion (33). By analyzing tumor fractions of *TP53* mutations in MM, it was found that this aberration appeared with higher frequency in subclonal, which implies preferential acquisition of these events later during clonal evolution of MM, as opposed to founder events. Analysis of co-occurrence of genomic events reveals that *TP53* mutations almost always occur after or simultaneously with allelic 17p13 deletion (44). Inversely, *TP53* mutations were found in about one third of MM patients carrying 17p deletion, with the tendency of increase to more than 50% in refractory disease (41, 45).

*TP53* biallelic inactivation is a driver of progression in MM. At diagnosis, biallelic events in terms of loss or mutation of *TP53* (called double hit myeloma) are detected in 3.7% of patients and represent a crucial marker of adverse prognosis for both PFS and OS, compared to wild-type or mono-allelic inactivation (46). In contrast, in a cohort of patients with relapsed MM, a *TP53* abnormalities was identified 45% of the patients, and a double-hit events del(17p)/TP53^mut^ or del(17p)/TP53^del^ were observed in 15% of the cases. Those patients had a worse outcome (47).

### Epigenomic Regulation

DNA methylation of the cytosines within CpG islands in promoter region of genes is an epigenetic modification known to decrease gene expression. Hypermethylation of the promoter of *TP53* has been reported in MM cell lines and the expression of p53 protein was increased after treating cells by demethylating agents (48). In the context of *TP53* haploinsufficiency in MM, it was shown that in cell lines without p53 protein expression the remaining allele was silenced by promoter hypermethylation (49). However, data on primary MM samples are lacking.

### MiRNAs Regulation of *TP53*

Regardless of low mutation and deletion rates of *TP53* in NDMM, the lack of functional protein is much more frequent than it would be expected based solely on detected genetic aberrations. This implies the existence of post-transcriptional mechanisms of regulation of p53 signaling in MM (50). The interaction between p53 and miRNAs is performed in both directions—p53 can act upon regulating transcription and maturation of several miRNAs, whilst miRNAs can perform either direct or indirect repression of p53 (51). The tumor suppressor miR-34 family is involved in repression of p53 pathway in MM through interaction with constituents of cell cycle and proliferation signaling pathways (52). MiR-34a expression is decreased in MM cells harboring 17p deletion/*TP53* mutation (53) and its promoter region is often hypermethylated in MM (54). MiR-125b and miR-504 are direct negative regulators of p53 as they bind to the 3′UTR of TP53 mRNA, and their overexpression lead to downregulation of the endogenous level of p53 protein, inhibiting apoptosis in human neuroblastoma, and lung fibroblast cells (55, 56). Deregulation of miR-125b in MM pathogenesis has also been confirmed (50).

### Post-translational Regulation

*MDM2* is overexpressed in several MM cell lines and PCL patients, inducing a down-regulation of p53 (57). *MDM2* gene localizes on 12q15. Gain if 12q is one of the most frequent copy number abnormalities in pan-cancer analysis (58, 59), however it is not often reported in MM.

## Targeting p53 in Multiple Myeloma

Although the clinical outcome of patients with MM was significantly improved with the introduction of novel therapeutic strategies such as new-generation proteasome inhibitors, immunomodulatory drugs, anti-CD38 antibodies, and more recently CAR-T cells, MM remains incurable.

Patients with a 17p13 deletion demonstrated significantly lower response rate to lenalidomide treatment compared to patients not bearing this abnormality (60). Moreover, combinational treatment with lenalidomide, Adriamycin, and dexamethasone showed similar results in relapsed patients with 17p13 deletion (61). These results emphasize the need for novel therapeutic approaches that would overcome the 17p13 deletion related resistance to conventional treatment.

**Figure 2:**
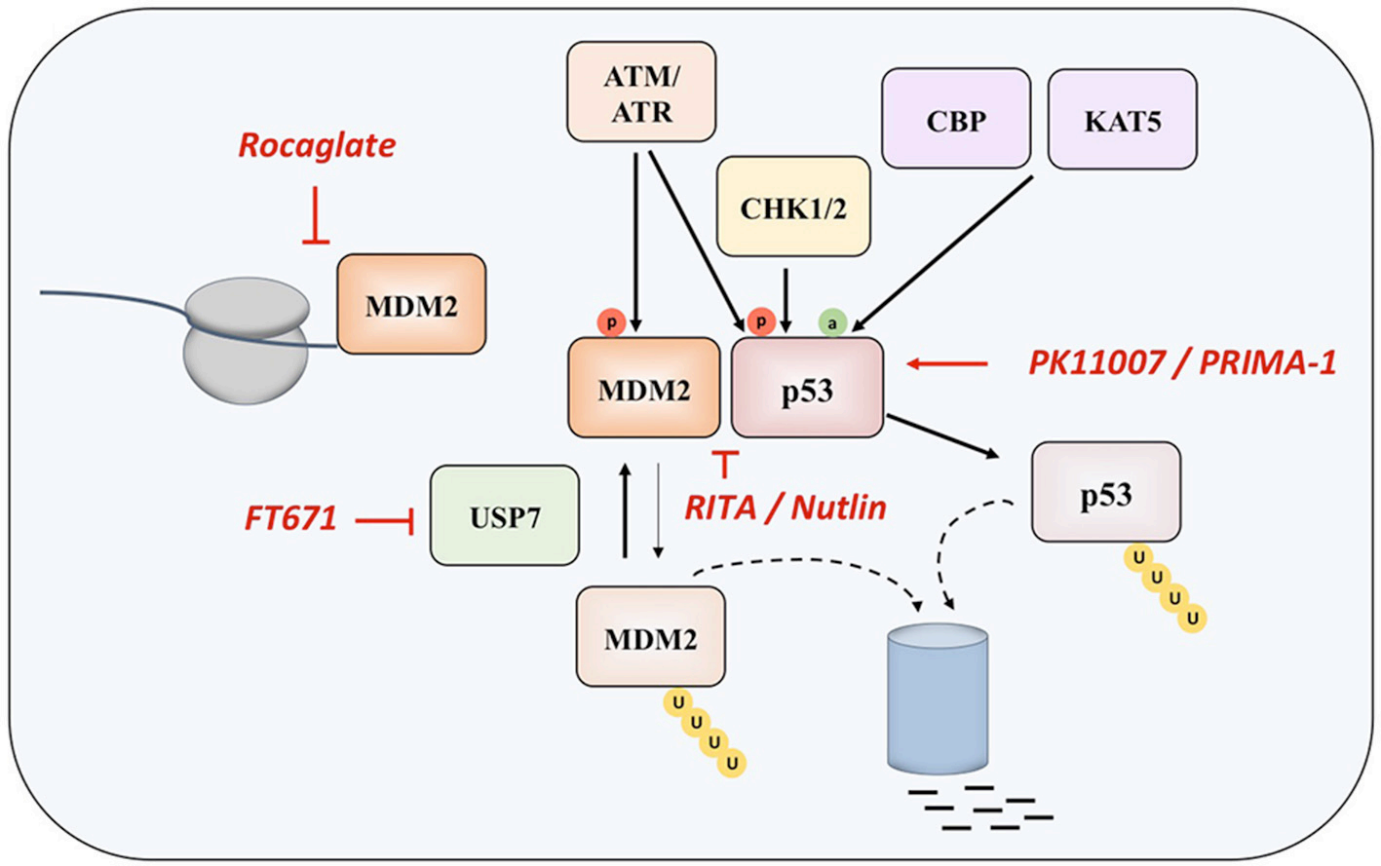
Interacting with *TP53* pathway. Different drugs interacting with MDM2 and p53 (in red).

Two main approaches in reestablishing normal function of p53 in MM and in cancer in general are based inhibiting the interaction of the protein with its negative regulators (such as MDM2 and MDM4), and on restoring the function of protein product of the mutated *TP53* gene (Figure 2).

Nutlin was detected as the first potential inhibitor of p53-MDM2 interaction, as it was found that prevents binding of p53 to MDM2, which results in stabilization, accumulation, and activation of p53 signaling in cancer cells. However, nutlin works only in cells with preserved p53 signaling pathway and wt p53 (62). Nutlin-3 was showed to have significant activity in MM in experiments performed *in vitro* and *ex vivo*, as well as in synergy with drugs such as melphalan, etoposide, and bortezomib (63, 64), however the drug induces resistance and clonal selection.

Another compound called RITA (Reactivation of p53 and Induction of Tumor Cell Apoptosis) binds directly to the N-terminal domain of p53, which reduces its affinity for MDM2 as a consequence of conformational changes in the structure of p53 protein (65), although later study questioned this mechanism of action (66). It appears that RITA sensitivity correlates with induction of DNA damage as resistant cells show increased DNA cross-link repair. Inhibition of FancD2 restores RITA sensitivity (67). In MM cells, which acquired resistance to other therapeutic approaches, RITA induced cell cycle arrest and apoptosis (68). However, when the efficacies of nutlin-3a and RITA were compared in a panel of HMCLs with different *TP53* statuses, it was found that, unlike nutlin-3a which exhibited toxicity only in HMCLs with wt-*TP53*, RITA killed 25% of HMCLs independently of the *TP53* status, and showed efficacy independently of the presence or absence of 17p deletion in primary patient samples, implying that RITA could be a therapeutic approach of choice in patients with *TP53* abnormalities, who showed resistance to current therapies (69).

When overexpressed in MM cells, *MDM2* maintains low levels of p53, thus inhibiting its tumor suppressive functions (70, 71). Recently, a compound CMLD010509, which is a synthetic analog of the rocaglate, was found to inhibit the oncogenic translation program in MM cells and in mouse models of MM, targeting MDM2 oncoprotein among other targets (72). This can be explained by the fact that rocaglates are inhibitors of translation initiation factors and that MDM2 has a short half-life, suggesting that these proteins are preferentially affected in case of low translational flux.

Another approach to interact with MDM2 is to inhibit the ubiquitin-specific protease 7 (USP7), which is as a deubiquitinase removing ubiquitin specifically from MDM2. The inhibition of UPS7 results in the degradation of MDM2 and leads to re-activation of p53 (73). The small molecule FT671 is a potent USP7 inhibitor with a potent effect *in vitro* and *in vivo* in various cancers.

In was demonstrated that *TP53*-deficient cells are highly sensitive to the inhibition of Ataxia-Telangiectasia Rad3-related (ATR), one of the kinases responsible for DNA damage response (DDR) (74). Likewise, MM cells lines bearing *TP53* mutation showed better response to ATR inhibition compared to *TP53* wild-type MM cell lines, implying that, in the subset of MM clones with increased replicative stress, and DNA damage, inhibition of ATR could be potentially exploited as a synthetic lethal therapeutic approach (75).

PRIMA-1 (p53 reactivation and induction of massive apoptosis) has been introduced as therapeutic agent with aim of restoring the original function of mutant p53, through obtaining the wt conformation of the protein (76). The more effective derivative, PRIMA-1^MET^, except restoring mutant p53, is also acting in a TP53-independent manner by inducing reactive oxygen species (ROS) in cancer cells, with a good efficacy in MM (77–81). In fact, the accumulation of mutant-p53 protein suppresses the expression of *SLC7A11*, a component of the cystine/glutamate antiporter, through binding to the master antioxidant transcription factor *NRF2*. This diminishes glutathione synthesis, rendering mutant-p53 tumors susceptible to oxidative damage (82).

Other therapeutic approaches have been recently investigated (83, 84). A novel compound PK11007, belonging to the class of selective thiol alkylators 2-sulfonylpyrimidines, was found to alkylate surface-exposed cysteines 182 and 277 of p53, which results in stabilization of its DNA binding domain *in vitro*. This compound is particularly effective in cancer cell lines with null or mutant p53 background (83). An innovative approach consists of functional screening of phage display libraries for peptides, which carry the potential for reactivation of the mutant p53 (84).

## Conclusion

*TP53* alterations are of adverse prognosis in MM as in cancer in general. Recent publications of next generation sequencing on large cohort of patients with MM have enabled to better define the incidence of TP53 alteration across the course of the disease. In MM, *TP53* is deregulated through different molecular mechanism, such as deletion17p, *TP53* mutations, MDM2 overexpression, methylation of *TP53* promoter, and deregulation of specific miRNAs. The knowledge of these molecular mechanisms of *TP53* pathway alteration has helped the development of therapeutic strategies to target this pathway. Different approaches are currently tested: blocking the interaction between MDM2 and p53 with small molecules such as Nutlin or RITA, targeting MDM2 by inhibiting translation initiation with rocaglate or by blocking its deubiquitination by USP7 inhibitors, and restoring the function of altered p53 proteins. All these therapeutic strategies are currently being tested and need to be further validated for clinical use. Considering that TP53 alterations increase during progression of MM and induce drug resistance, it will be necessary to interact with *TP53* pathway to be able to cure MM, or at least develop strategies that do not select *TP53* subclones.

## Author Contributions

KJ, GE, JD, AW, ZV, MF, PC, TF, BQ, and SM wrote the manuscript, generated the figures, and revised the review.

### Conflict of Interest Statement

The authors declare that the research was conducted in the absence of any commercial or financial relationships that could be construed as a potential conflict of interest.
